# Alexithymia and emotional regulation: A cluster analytical approach

**DOI:** 10.1186/1471-244X-11-33

**Published:** 2011-02-23

**Authors:** Jie Chen, Ting Xu, Jin Jing, Raymond CK Chan

**Affiliations:** 1 Department of Maternal and Child Health, school of Public Health, Sun Yat-Sen University, Guangzhou, PR China; 2 Neuropsychology and Applied Cognitive Neuroscience Laboratory; Key Laboratory of Mental Health, Institute of Psychology, Chinese Academy of Sciences, Beijing, PR China; 3 Mental Health Education & Counseling Center, Sun Yat-sen University, Guangzhou, PR China; 4 Graduate School, Chinese Academy of Sciences, Beijing, PR China

## Abstract

**Background:**

Alexithymia has been a familiar conception of psychosomatic phenomenon. The aim of this study was to investigate whether there were subtypes of alexithymia associating with different traits of emotional expression and regulation among a group of healthy college students.

**Methods:**

1788 healthy college students were administered with the Chinese version of the 20-item Toronto Alexithymia Scale (TAS-20) and another set of questionnaires assessing emotion status and regulation. A hierarchical cluster analysis was conducted on the three factor scores of the TAS-20. The cluster solution was cross-validated by the corresponding emotional regulation.

**Results:**

The results indicated there were four subtypes of alexithymia, namely extrovert-high alexithymia (EHA), general-high alexithymia (GHA), introvert-high alexithymia (IHA) and non-alexithymia (NA). The GHA was characterized by general high scores on all three factors, the IHA was characterized by high scores on difficulty identifying feelings and difficulty describing feelings but low score on externally oriented cognitive style of thinking, the EHA was characterized by high score on externally oriented cognitive style of thinking but normal score on the others, and the NA got low score on all factors. The GHA and IHA were dominant by suppressive character of emotional regulation and expression with worse emotion status as compared to the EHA and NA.

**Conclusions:**

The current findings suggest there were four subtypes of alexithymia characterized by different emotional regulation manifestations.

## Background

Alexithymia has been a familiar conception as "no words for feeling" in psychiatry and psychosomatic medicine since it was first termed by Sifneos [1]. Now its definition is more explicitly refined with five dominant features: (1) difficulty in identifying one's emotion; (2) difficulty in describing self feelings verbally;(3) a reduction or incapability to experience emotions;(4) an absence of tendencies to image one else's emotion, or an externally oriented cognitive style; and (5) poor capacity for fantasize or symbolic thought [2]. Alexithymia refers to a specific disturbance in emotional processing, especially reduced capabilities in verbalizing and realizing emotion. Longitudinal study also suggested that alexithymia was significantly associated with the severity of depression [3], anxiety [4] and schizophrenia [5]. The prevalence rate of alexithymia is significantly higher in patients with psychosomatic disorders, such as eating disorder[6], fibromyalgia syndrome[7] and low-back pain[8], than control groups.

Researchers [9,10] found alexithymia overlaps with various dimensions like external locus of control and irrational beliefs, except impulsiveness, of the Five-Factor Model (FFM) of personality in an undergraduate student sample. It has been speculated that alexithymia is a cognitive state of externally oriented thinking with an emotional instability and unsecure performance in controlling stressful situation. However, alexithymia has also been criticized whether it is an affect-deficit disorder (state-orient) or a continuous personality variable (trait-orient). Tolmunen et al. [11] considered alexithymia as a stable personality trait in general. Their 11-year follow-up study also suggested that alexithymia might increase vulnerability to depressive symptoms[11]. Honkalampi [12] further demonstrated that depressive symptom might act as a mediator between alexithymia and psychiatric morbidity. Parker and Mattila used taxometric analysis to synthesize several studies about alexithymia in large sample pools including general population and psychotic patients [11,12]. These findings suggest that "aleixhtymia is not a discrete affect deficit type of person but represents 'the lower tail' of an emotion processing ability that is continuously distributed in the general population" [11].

The purpose of this study was to examine whether there might be subtypes of alexithymia characterized by different behavioural manifestations. In so doing, the current study adopted a cluster analytical approach to examine whether there were natural grouping of people characterized by different psychological features associating with alexithymia. Cluster analysis is a statistical procedure for determining cases can be placed into groups if they share properties in common, while the cases in different clusters are as dissimilar as possible. It was hypothesized that there were various subtypes of alexithymia characterized by different psychological features associating with alexithymia.

Individuals on various level of alexithymia would adopt different ways to express and regulate their emotion. The higher alexithymia groups would perform more serious level of depressive or anxious emotional status and more possible to adopt improper regulation strategy.

## Method

### Participants

1788 college students (freshmen and sophomore) were recruited from three regional universities in Guangzhou, south China. 1071 were males and 616 were females, aged 20.44 ± 1.40 years and 20.51 ± 1.39 years respectively, 101 individual did not mention their gender or age. Economic status was also recorded by a multiple-choice question in the checklist for monthly income per person a month. Among all subjects, 110 individuals did not mention their economic status.

All subjects were literarily informed the aim of current study was to examine about psychological status of Chinese youngsters and were voluntarily attended this study. All of them would receive a feedback on the assessment results via email. This study was approved by the ethics committee of the Sun Yat-Sen University.

### Measurements

Alexithymia was assessed by the 20-item Toronto alexithymia scale (TAS-20) to assess the severity of alexithymia[13,14]. It is a 20-item self-report instrument rated on a 5-point Liker-type scale ranging from 1 (strongly disagree) to 5 (strongly agree). Total scores range from 20 to 100, with higher scores indicating higher level of alexithymia. Within the five criteria, the TAS-20 consists of 3 factors: difficulty identifying feelings (DIF); difficulty describing feelings (DDF); externally oriented cognitive style of thinking (EOT). The Chinese version has been shown with having the same factor structure of the original version and has been associated with good internal consistency [15], which was adopted in this study. The Cronbach's α coefficient of it was 0.83, the test-retest reliability coefficient was 0.87, the mean inter-item correlation coefficients ranged from 0.13 to 0.32, the correlate on coefficients of the three factors with the total scale score ranged from 0.72 to 0.82, the correlation coefficients among the three factors ranged from 0.29 to 0.54 [16].

Emotion expression tendency was assessed by the Chinese version of the Emotional Expressivity Scale (EES) [17]. It is a 17-item self-report assessing the ability to express emotion rated on a 6-point Liker-type scale (1 = never true to 6 = always true). There were two factors in the Chinese version, namely the emotional suppression and emotional expression [18]. The Cronbach's alpha coefficient for the total scale showed a high internal consistency reliability of 0.816. Cronbach's alphas for the two factors were 0.84 and 0.78 respectively indicating adequate internal consistency [18]. Higher total score reflects a higher ability to express emotion, higher expressive factor score means higher intention to express, but lower suppressive factor score means higher inclination to control emotion.

Emotion Regulation Questionnaire (ERQ) [19] was used to measure emotion regulation. The ERQ is a 10-item checklist capturing two commonly used emotion regulation strategies, i.e., reappraisal and suppression. Reappraisal refers to the use of methods changing the way of thinking about a potential emotional event, whereas suppression refers to the adoption of regulation to suppress when facing the same emotional event. Subjects were required to rate their respond to a 7-point Liker-type scale (1 = totally disagree to 7 = totally agree) on their usual ways of emotional regulation. The test-retest reliability and a coefficient of Chinese version of ERQ were 0.82 and 0.85 for reappraisal dimension, were 0.79 and 0.77 for suppression [20]. Higher score indicates a higher tendency to adopt such strategy. Reappraisal strategy was thought to be a more appropriate way to regulate emotion than suppression one.

Depression was measured with the Beck depression inventory (BDI) [21,22]. It is a 21-item scale to assess depression problems with higher score representing more depression tendency. The current study adopted the Chinese version of BDI, which Cronbach's alpha coefficient was found to be 0.85 [23].

Anxiety was assessed with the Chinese version of the state portion of State-Trait Anxiety inventory (STAI-T) [24]. This is a self-reported scale containing 20 items assessing level of anxious status rated on a 4-point Liker-type scale (1 = never to 4 = always). The Cronbach's alpha coefficient showed a high internal consistency reliability was 0.81. The higher score refers a more serious anxious state.

### Data analysis

The Statistical Package for Social Sciences (SPSS) 15.0 (SPSS Inc, Chicago, IL, USA) was used for all statistical analyses reported.

Independent sample t-tests were conducted to analyze the gender effect on the total scores of TAS-20. ANOVA was conducted to evaluate the potential effect of economic status upon the total scores of TAS-20, whereas correlation analyses were used to explore any association of education and age with the TAS-20 scores.

Cluster analyses were conducted in two phases. First, a hierarchical cluster analysis was conducted using the 3 factors: difficulty identifying feelings; difficulty describing feelings; externally oriented cognitive style of thinking scores of TAS-20 as the clustering variables and the between-group linkage method with a squared Euclidean distance measure to discriminate clusters. Second, the cluster solution was validated with analysis of variance (ANOVA) on scores of Emotional Expressivity Scale with its subscales, Emotion Regulation Questionnaire, Beck depression inventory and the state portion of State-Trait Anxiety inventory of the identified groups.

## Results

No significant difference was found in the total mean TAS-20 scores between boys and girls (49.63; SD, 8.70 vs 48.96; SD, 8.60; p = 0.13). Age was significantly correlated the total mean TAS-20 (r = 0.05, p = 0.04), whereas there was no significant association between education and TAS-20 total score (r = 0.04, p = 0.11). No significant different in the total mean TAS-20 scores between economic status was found (F = 2.06, p = 0.08). Given the effect of age upon the TAS-20 score was negligible, it was not controlled for subsequent analyses between cluster comparisons.

Table 1 shows there were four subtypes of alexithymia groups, namely extrovert-high alexithymia (EHA), general-high alexithymia (GHA), introvert-high alexithymia (IHA) and non-alexithymia (NA). The extrovert-high alexithymia (EHA) group was characterized a relative high in externally oriented cognitive style, regular scores in difficulty identifying feelings and difficulty describing feelings and contained most of the cases (77.3%). The general-high alexithymia (GHA) group was characterized by a high score of every factor of alexithymia. The introvert-high alexithymia (IHA) was characterized by a significant high score in difficulty identifying feelings and difficulty describing feelings, which referring to self emotional experience, but relative low score in externally oriented cognitive style of thinking scores. Finally, the non-alexithymia (NA) group was characterized a general low score of alexithymia problems.

**Table 1:** Description of subtypes of alexithymia

Subtype	Extrovert-high alexithymia group	General-high alexithymia group	Introvert-high alexithymia group	Non-alexithymia group	F	P
			
	N = 1382 (77.3%)	N = 43 (2.4%)	N = 47 (2.6%)	N = 316 (17.7%)		
			
	Mean ± SD	Mean ± SD	Mean ± SD	Mean ± SD		
Alexithymia characteristics						
Score of Difficulty Identifying Feelings factor of TAS-20	17.66 ± 3.20	27.47 ± 3.81	24.68 ± 2.55	11.37 ± 2.76	597.81	<0.001**
Score of Difficulty Describing Feelings factor of TAS-20	13.56 ± 2.42	17.49 ± 2.93	15.13 ± 3.89	9.95 ± 2.36	236.07	<0.001**
Score of Externally Oriented cognitive style of Thinking factor of TAS-20	20.39 ± 3.00	24.33 ± 3.78	15.00 ± 2.45	15.56 ± 3.34	278.71	<0.001**
Total score of TAS-20	51.62 ± 6.12	69.28 ± 4.92	54.81 ± 5.47	36.87 ± 4.47	718.74	<0.001**
Demographic data						
Age (years)	20.49 ± 1.28	21.10 ± 1.08	20.40 ± 1.36	20.39 ± 1.19	3.20	0.02
Education (years)	14.81 ± 1.09	15.23 ± 1.37	14.94 ± 1.09	14.70 ± 1.02	3.85	0.01

Note: TAS-20 = 20-itemToronto alexithymia scale, *P < 0.05, **P < 0.01 (two-detailed)

The four clusters did not differ significantly in terms of gender propotion (Chi-square = 0.84, df = 3, p = 0.84) and economic status (Chi-square = 16.18, df = 12, p = 0.17). However there showed significant difference in terms of age (t = 3.20, p = 0.02) and education (t = 3.85, p = 0.01).

An ANOVA showed that the four subtypes of alexithymia differed significantly in terms of emotional status, emotional expression and regulation as our estimation (Table 2). The general-high alexithymia (GHA) and introvert-high alexithymia (IHA) groups showed dominant higher level of depression and anxious than the extrovert-high alexithymia (EHA) and non-alexithymia (NA) groups. The GHA demonstrated significantly higher scores on suppressive factor in Emotional Expressivity Scale (EES) and Emotion Regulation Questionnaire (ERQ). The introvert-high alexithymia (IHA) group also demonstrated higher scores in suppressive tendency in expressing emotion but adopting more reappraisal strategies in regulating emotion than the GHA group. The non-alexithymia (NA) exhibited the highest will to express their emotions and to choose more reappraisal strategies to regulate their emotions, and was associated with the least depressive and anxiety problems. The extrovert-high alexithymia (EHA) were modest between NA and GHA groups.

**Table 2:** Emotional status, emotional expression and regulation traits among subtypes of alexithymia (ANOVA)

	Extrovert-High Alexithymia group (N = 1382)	General-High Alexithymia group (N = 43)	Introvert-High Alexithymia group (N = 47)	Non-Alexithymia group (N = 316)	F	p	Between groups comparisons
				
	Mean ± SD	Mean ± SD	Mean ± SD	Mean ± SD			
Emotional Expressivity Scale							
Total score of Emotional Expressivity Scale	60.26 ± 9.83	54.33 ± 12.29	58.45 ± 14.45	62.60 ± 11.48	10.33	<0.001**	4>1 > 2*
Suppression factor of Emotional Expressivity Scale	43.71 ± 7.37	38.16 ± 10.49	41.17 ± 9.95	45.81 ± 8.42	16.58	<0.001**	4>1 > 2*, 4 > 3*
Expression factor of Emotional Expressivity Scale	16.54 ± 4.42	16.16 ± 5.72	17.28 ± 5.91	16.79 ± 4.94	0.72	0.541	No group difference
Emotion regulation questionnaire							
Reappraisal factor of Emotion regulation questionnaire	29.30 ± 5.35	26.09 ± 7.61	30.49 ± 5.94	31.98 ± 6.40	25.82	<0.001**	4 > 1,2**, 3 > 2*
Suppression factor of Emotion regulation questionnaire	14.67 ± 3.96	16.74 ± 5.10	14.57 ± 5.10	12.82 ± 4.69	21.75	<0.001**	1,2 > 4*
Emotional status							
score of Beck Depression Inventory	7.37 ± 7.61	14.79 ± 10.53	12.06 ± 9.98	3.27 ± 4.70	51.87	<0.001**	2,3>1 > 4*
Trait score of Stat and Trait anxious inventory.	43.42 ± 7.11	51.16 ± 12.34	48.62 ± 10.31	36.77 ± 7.62	99.11	<0.001**	2,3>1 > 4*

Note: *P < 0.05, **P < 0.01 (two-detailed)

## Discussion

The major findings of this study showed there were four subtypes of alexithymia and were consistent with previous studies. For example, Vorst and Bermond [25] suggested that there were two types of alexithymia characterized by the emotional and cognitive factors of the Bermond-Vorst Alexithymia Questionnaire (BVAQ)[26]. They proposed that Type I alexithymia is characterized by a low degree of conscious awareness of emotional arousal and a low degree of emotion accompanying cognitions; whereas Type II alexithymia is characterized by a normal or high degree of conscious awareness of emotional arousal together with a low degree of emotion accompanying cognitions. Our cluster analysis showed that there were 4 subtypes of participants associating with different degrees of alextithymia in the college students, namely the extroverted-high alexithymia (EHA), general-high alexithymia (GHA), introversive-high alexithymia (IHA) and non-alexithymia (NA). The GHA was characterized by a general low profile of emotional cognition including identifying, describing self emotion and external imagination. The general-high alexithymia (GHA) was similar to Type I alexithymia mentioned by Vorst. The introversive-high alexithymia (IHA) was dominant by low arousal of self emotional experience but normal ability of externally oriented thinking style, which was very similar to Type II. The extroverted-high alexithymia (EHA) is characterized by a normal range of self emotional arousal and a profile score of externally oriented thinking style. These features were very similar to those of Type II alexithymia.

Validation of the cluster solution suggested that these subtypes of alexithymia were characterized by different emotional expression and regulation abilities. The general-high alexithymia (GHA) and introversive-high alexithymia (IHA) were characterized by poorer emotional regulation and expression with worse emotion status. In more details description, the extroverted-high alexithymia (EHA) seemed to be modest in emotion status, with emotion regulating more efficiently as compared to the general-high alexithymia (GHA). These results suggest the potential functional outcome of these different subtypes. Mattila [27] found that individuals with alexithymia showed significantly lower satisfaction to many dimensions of general life than individuals without alexithymia. Our current findings also showed that individuals with general-high alexithymia (GHA) and introversive-high alexithymia (IHA) tended to show less effective ways to regulate their emotion and might face more stress in their social life than other groups. These findings highlight the need for timely and appropriate psychological counseling for these individuals. The characteristics associated with the different clusters of individuals with alexithymia suggest that regulation ability of alexithymia may require different intervention regimes to protect or maintain their own emotional regulation and expression.

It should be noted that the EHA cluster includes the 77.3% of the sample. The EHA cluster shows alterations mostly on externally oriented cognitive style of thinking (EOT). Some studies showed difficulty identifying feelings (DIF) and difficulty describing feelings (DDF) had good internal reliability but not EOT [28]. Some researcher thought EOT dimension showed different development paths companying with DDI and DDF [29]. Moreover, it should also be cautious that the clusters we found in the current study may not be stable at all in time and/or it is an artefact due to the instruments that have been used by the current study. It should be necessary to reassess the students to evaluate validity. Without a longitudinal approach any speculation in the discussion should be shown just as an hypothesis to be confirmed. Our results indicated that most of the cases were clustered into extroverted-high alexithymia (EHA) and suggested alexithymia might be a general phenomenon of an emotion processing ability distributed in the general population. More investigations were needed to clarify the relation of alexithymia and personality.

The current study has several limitations. First, participants were recruited from a convenient sample pool was the main limitation of this study. Whether these cluster groups could discovered in more broad population needs future study adopted a more rigorous epidemiological approach to improve its representativeness. Second, the findings were based on subjective self-report measures. More rigorous methodologies adopting experimental designs or neurophysiological approaches such as ERP or imaging paradigms should be enforced in the near future in order to validate potential differential neural bases of these subtypes of alexithymia. Finally, the current cross-sectional design could not examine the stability of the cluster solutions across different time points. Future study should adopt a longitudinal design to test the stability of the cluster solution.

## Conclusions

The current findings suggest there were four subtypes of alexithymia characterized by different emotional regulation manifestations.

## Abbreviations

ANOVA: analysis of variance; BDI: Beck Depression Inventory; BVAQ: Bermond-Vorst Alexithymia Questionnaire; DDF: difficulty describing feelings; DIF: difficulty identifying feelings; EES: Emotional Expressivity Scale; EHA: extrovert-high alexithymia; EOT: externally oriented cognitive style of thinking; ERP: Event-related Potential; ERQ: Emotion Regulation Questionnaire; FFM: Five-Factor Model; GHA: general-high alexithymia, IHA: introvert-high alexithymia; NA: non-alexithymia; SPSS: Statistical Package for Social Sciences; STAI-T: Trait portion of the State-Trait Anxiety inventory; TAS-20: 20-item Toronto alexithymia scale.

## Competing interests

The authors declare that they have no competing interests.

## Authors' contributions

JC designed the study, collected and analyzed data, and wrote the first draft of the manuscript. RCKC conceived and designed the study, and wrote the first draft of the paper. TX analyzed data and participated in writing up the manuscript. JJ participated in writing up the manuscript. All authors read and approved the final manuscript

## Pre-publication history

The pre-publication history for this paper can be accessed here:

http://www.biomedcentral.com/1471-244X/11/33/prepub
